# Hybrid surgery techniques for the treatment of in-stent restenosis after 5 years of femoral artery self-expanding bare-metal stent implantation: A case report

**DOI:** 10.1097/MD.0000000000029042

**Published:** 2022-03-11

**Authors:** Jianguo Zhou, Guosong Zha, Guosheng Qian

**Affiliations:** Department of General Surgery, Linping Campus, The Second Affiliated Hospital of Zhejiang University School of Medicine, Hangzhou, China.

**Keywords:** arteriosclerosis obliterans, femoral artery, in-stent restenosis

## Abstract

**Rationale:**

Lower extremity arteriosclerosis obliterans (ASO) disease is caused by the formation of atherosclerotic plaque in the femoral artery, which causes the stenosis and occlusion of lower legs, and then leads to chronic limb ischemia. Stent intervention is the most common treatment for ASO in the lower extremities, although there is a risk of overstretching or fracturing the stent, resulting in stent rupture. We provide a unique method for treating stent rupture.

**Patient concerns:**

A 79-year-old male presented with intermittent claudication of the left lower limb for 6 months. Five years ago, a stent was placed in the lower extremity femoral artery. According to the examination, the stent suffered a modest torsional fracture.

**Diagnosis:**

The case was diagnosed with lower extremity ASO.

**Interventions:**

We performed a combination of femoral endarterectomy and interventional surgery.

**Outcomes:**

Blood flow was restored after the hybrid operation has been used to treat arterial stenosis in the lower limbs.

**Conclusion:**

Integrating vascular interventional surgeries can shorten surgical procedures time and increase success rates.

## 1 Introduction

Arteriosclerosis obliterans of the lower limbs are becoming more common as society's overall lifestyles improve and the population ages. Vascular stents have long been the prefered treatment for lower limb arteriosclerosis because of their excellent therapeutic efficiency and little surgical trauma. While vascular stenting has been successfully implanted, postoperative complications have always been a significant issue, compromising the therapeutic efficacy and posing a direct threat to patients’ lives.

Endovascular therapy has become a standard treatment for peripheral artery disease. When compared to typical percutaneous endovascular angioplasty, self-inflating metal stent insertion considerably boosted the femoral artery patency rate.^[1–3]^ Restenosis within the stent, on the other hand, is a well-known complication following stent placement.^[4,5]^ In-stent restenosis, which leads to stent re-occlusion, has long been recognized as one of the most prevalent causes of postoperative complications.^[6]^ The current research on the management of in-stent restenosis is discordant. This paper describes a case of stent obstruction caused by in-stent restenosis and treated utilizing hybrid surgical techniques.

## 2 Case presentation

**Figure 1:**
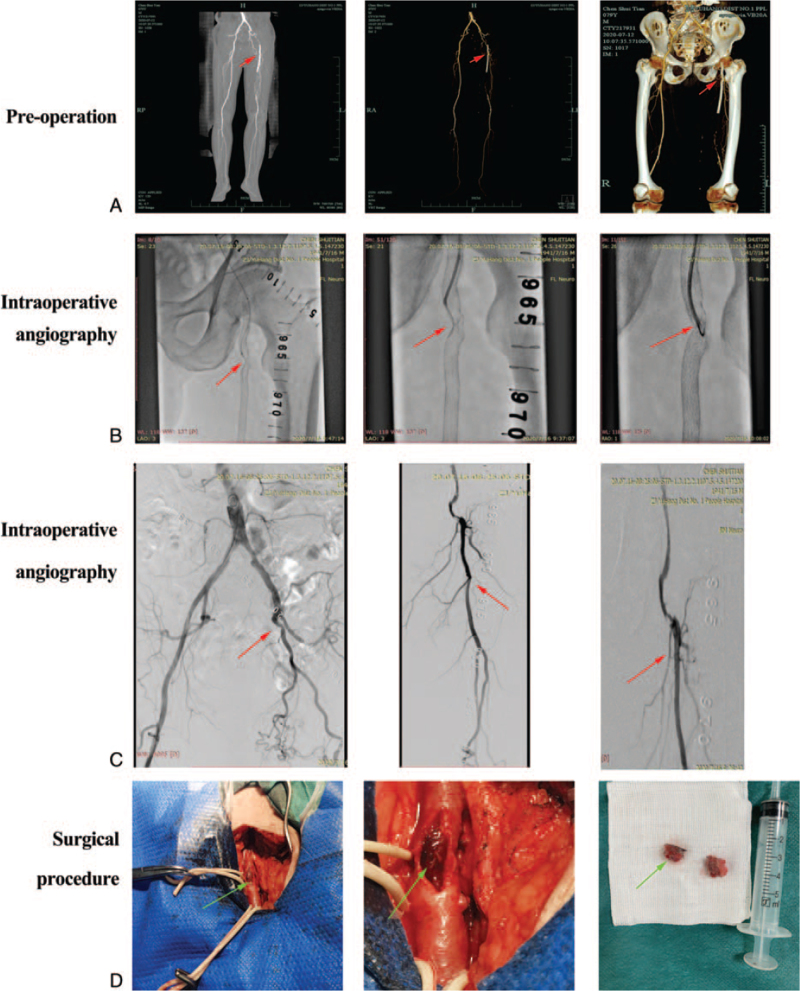
(A) Preoperative contrast-enhanced CT scan image and 3D reconstruction of the femoral artery of the lower extremity. (B) In-stent restenosis visible from intraoperative angiography. (C) Intraoperative angiography of the left side of the external iliac artery, femoral artery and femoral artery occlusion (red arrow). (D) The stenosis and rupture of the femoral artery stent may be seen once the femoral artery has been exposed.

Patient data: A 79-year-old male patient had intermittent claudication of the left lower limb for about 6 months, with a claudication distance of about 30 cm. Five years ago, the patients had a femoral artery stenting of the left lower leg stent implantation. According to a contrast-enhanced CT scan of the ischemic zone, the preceding stent had a local torsion fracture, which caused the claudication (Fig. 1A). In the risk factors assessment, the patient also had hypertension.

Surgical treatment: Anesthesiologist administering general anesthesia to the patient after endotracheal intubation. Puncture from the right femoral artery through the right common iliac artery to the left common iliac artery, and in-stent restenosis visible from first intraoperative angiography (Fig. 1B). Then Intraoperative angiography of the left side of the external iliac artery, femoral artery and femoral artery occlusion, and deep arterial patency (Fig. 1C). Proximal openings in the original stents, we block blood flow by 7 ∗ 60 ARMADA balloon. After the lumen was blocked, the left leg segment was incised, and exposed the femoral artery as well as the surrounding either deep or shallow artery were also exposed. After incising open the left femoral artery, we discovered that the previous stent was twisted and fractured (Fig. 1D). The twisted stent was then removed, followed by the tunica intima being stripped. After the distal end of the original stent was expanded with a biliary tract probe, the guide wire was sent to the distal end of the preceding stent, and the blood vessel wall of the left femoral artery was sutured. When the balloon was withdrawn to clear the blockage, there was no bleeding at the incision site of the left femoral artery. Through the blockage of the original stent and the distal end of the stent, a guided wire catheter was utilized to enter the popliteal artery. After determining the exact lumen, the 4∗150 mm and 5∗150 mm Mustang balloons were used to dilate the external iliac artery's occluded vascular segment, followed by 7∗60 mm ARMADA balloons to entirely dilate the occlusion segment. Finally, a MUSTANG balloon with a diameter of 5∗150 mm was employed to widen the vessel's superficial section (adjacent to the original stent). The outcome of this angiographic was excellent. After the operation, dual anti-platelets (Aspirin & Polyvir) and vasodilator (Kaina) were given. One month after the procedure, blood flow was unobstructed at the location where the original stent was removed, and local stenosis was detected at the place where the prior stent was removed. Three months following surgery, the stent was completely blocked underneath the previous stent's placement, and blood flow in the stent's distal popliteal artery was adequate.

Secondary surgical treatment: Angiography under local anaesthetic revealed that the left iliac artery, common femoral artery, and deep femoral artery all had continuous blood flow. On the other hand, the new stent distal to the old stent was completely obstructed. The blood flow through the popliteal artery was continuous at the stent's distal end. The balloon section, which was roughly 5∗300 mm in diameter, was checked, and the angiographic result showed that blood flow was still present. According to a contrast-enhanced CT scan of the lower limb arteries 1.5 months after the surgery, blood flow in the lower limb arteries remained constant.

**Figure 2:**
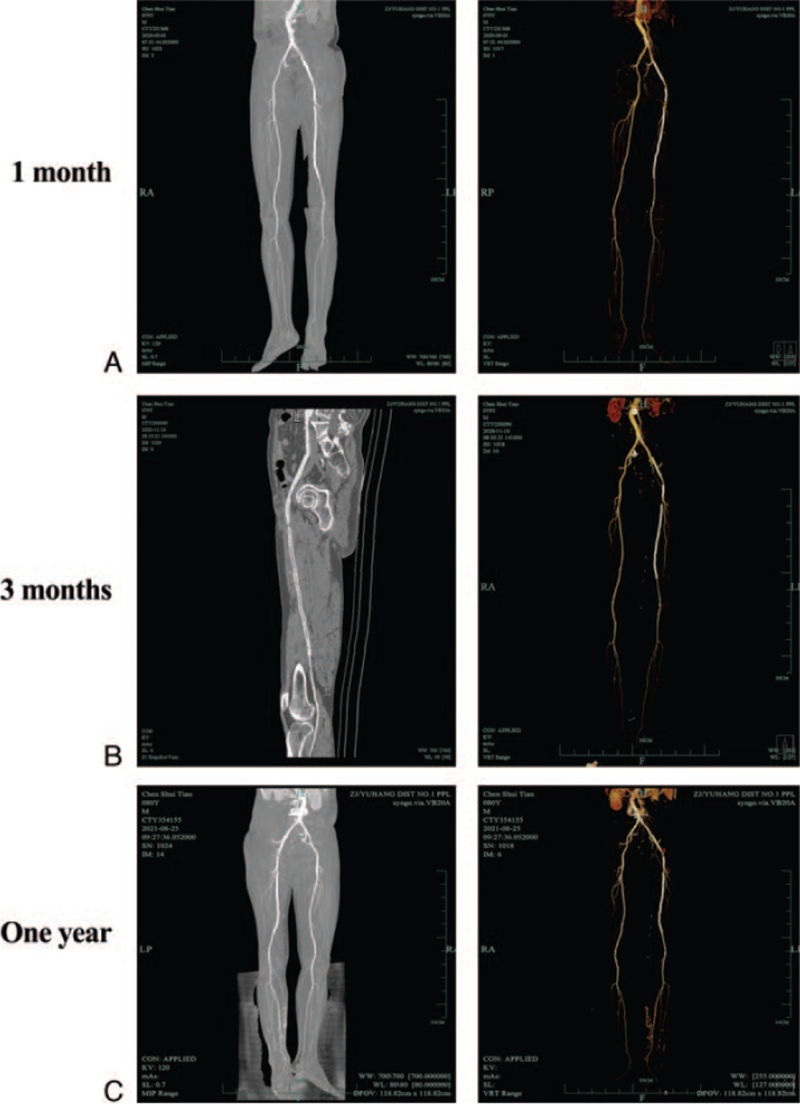
C D&E The post-operative CT images at 1 month, 3 months, and 1 year after dual surgeries, the results of successful cannulation at the lower extremity arteries.

Outcome and follow-up: At 1 month, 3 months, and 1 year after surgery, we used a contrast-enhanced CT scan and 3D reconstruction to analyse the patient's improvement (Fig. 2A, B&C). The femoral arteries showed no signs of stenosis, indicating that the treatment was effective.

## 3 Discussion

Lower limb artery stenting is a conventional therapy for peripheral arteria disease that has been shown to have a greater long-term patency rate than traditional percutaneous transluminal angioplasty. However, stent rupture is a common complication following stenting of the lower leg artery. According to Jaff et al., stent rupture in the superficial femoral artery may be categorized into 5 subtypes.^[7]^ A Type I stent rupture involves just 1 stent. A Type II stent rupture involves many stents rupturing in diverse sites. In Type III, a series of stent fractures culminated in a complete transverse fracture with no displacement. A Type IV stent rupture resulted in a complete transverse fracture with stent displacement, and Type V was a spiral fracture, also known as torsion fracture. Stent rupture can result in catastrophic modifications in the target vasculature and is a common cause of in-stent restenosis (ISR), particularly in lengthy segments where pseudoaneurysms might form.^[8,9]^

ISR refers to the narrowing of the luminal caliber of the stent owing to the development of stenosis inside the stent itself. ISR should be differentiated from stent compression. The treatment options available at present include balloon angioplasty (hyperdilation or isodilation), laser ablation, atherectomy, and Z-stent placement.^[11]^ There is no common protocol for the treatment of ISR following the implantation of a peripheral arterial disease stent in the lower extremities. For certain ISRs that aren’t too severe, complete endovascular therapy may be an option.^[10]^ However, severe and serious ISRs require open bypass surgery, which includes a substantial portion of surgical trauma.^[12]^ When our initial attempt at endovascular therapy failed, we turned to open surgery in combination with endovascular treatment. This method not only expedites the treatment but also resolves complicated endovascular interventions and improves the success rate of the operation.

In an endovascular combined open surgery, our experience indicates that vascular mesh and artificial vessels should be prepared prior to operation. When suturing the leftover vascular membrane after stent removal is problematic, vascular mesh or artificial vessels may be used to avoid artery stenosis at the stitching segment. Drug-coated balloons for occlusive stents may be more beneficial in the long run than conventional balloons, although they are more costly.^[13]^

## Author contributions

**Conceptualization:** Jianguo Zhou.

**Data curation:** Guosheng Qian& Guosong Zha

**Formal analysis:** Jianguo Zhou.

**Methodology:** Jianguo Zhou.

**Resources:** Jianguo Zhou.

**Writing – original draft:** Jianguo Zhou.

**Writing – review & editing:** Jianguo Zhou.
